# Clinical features and trends of severe paediatric group A streptococcal infections in Japan in the post-COVID-19 pandemic era

**DOI:** 10.1017/S0950268826101162

**Published:** 2026-02-19

**Authors:** Ayumi Tada, Shogo Otake, Taizo Kusano, Tadashi Hoshino, Masayoshi Shinjoh, Taizo Wada, Masaaki Mori, Hiroyuki Moriuchi, Naruhiko Ishiwada

**Affiliations:** 1 Department of Infectious Diseases, Medical Mycology Research Center, Chiba University, Japan; 2 Department of Pediatrics, Kobe University Graduate School of Medicine School of Medicine, Japan; 3 Division of Infectious Diseases, Chiba Children’s Hospital, Japan; 4 Department of Pediatrics, Keio University School of Medicine, Japan; 5 Department of Pediatrics, Kanazawa University, Japan; 6 Department of Lifelong Immunotherapy, Institute of Science, Japan; 7 National Research Center for the Control and Prevention of Infectious Diseases, Nagasaki University, Japan

**Keywords:** invasive group A Streptococcus, streptococcal toxic shock syndrome, empyema, children, Japan

## Abstract

A global increase in severe group A *Streptococcus* (GAS) infections has been reported following the COVID-19 pandemic, but data from Asia remain limited. We examined epidemiology and clinical characteristics of severe paediatric GAS infections across 86 Japanese hospitals, focusing on patients under 18 years of age, hospitalized between 1 January 2019 and 31 March 2024. Severe GAS infection was defined by the isolation of GAS from sterile sites, or from non-sterile sites under specific conditions, such as streptococcal toxic shock syndrome (STSS). A total of 83 cases were analysed. Cases increased from the summer of 2023, exceeding pre-pandemic levels. The median age was 4 (interquartile range: 1–8) years, with the highest number among 1-year-olds. No cases were reported in Hokkaido, northern Japan. Only 6% (5/83) of the cases had preceding GAS pharyngitis. Pneumonia was the most prevalent diagnosis (25%), with 76% of these cases being complicated by empyema, often necessitating intensive care or surgical intervention. Only 17% (14/83) of cases were reported as STSS in Japan’s national surveillance system. This study represents the first multicentre nationwide hospital-based investigation of severe paediatric GAS infections in Japan, identifying the recent increase in cases, thereby highlighting limitations of current STSS-based surveillance.

## Introduction

Group A streptococcal (GAS) infections encompass a wide spectrum of diseases, ranging from mild conditions, such as pharyngitis and scarlet fever, to life-threatening invasive infections. Invasive GAS (iGAS) infections affect both children and adults, presenting with diverse clinical manifestations, including pneumonia with empyema, septic arthritis, streptococcal toxic shock syndrome (STSS), and necrotizing fasciitis. Despite appropriate antimicrobial therapy, the mortality rate remains high, accounting for more than 160000 deaths annually, thereby posing a significant clinical and public health challenge globally [1].

During the COVID-19 pandemic, iGAS infections declined worldwide between 2020 and 2021. However, since late 2022, a rapid increase in iGAS infections and associated mortality has been reported in several European countries, North America, some regions in South America, and Australia, particularly among children below 10 years of age [2–10]. In response, both the World Health Organization and the Centers for Disease Control and Prevention issued public health alerts in December 2022 [11, 12]. However, large-scale epidemiological studies focusing on severe GAS infections in children in Asian countries remain limited, and the epidemiological trends before and after the COVID-19 pandemic are not well understood.

Although many countries conduct surveillance for iGAS infections, defined as GAS isolation from sterile sites, the National Epidemiological Surveillance of Infectious Diseases (NESID) in Japan focuses solely on STSS, not on iGAS infections overall [13]. According to the surveillance data, the incidence of STSS in Japan decreased during the COVID-19 pandemic but subsequently increased following the relaxation of infection control measures owing to the reclassification of COVID-19 in the Infectious Disease Control Law in May 2023. The National Institute of Infectious Diseases, currently a part of the Japan Institute for Health Security, reported an increase in both the number of STSS cases and the percentage of fatalities at the time of notification since July 2023, surpassing pre-pandemic levels, particularly among individuals below 50 years of age [14]. Generally, the frequency with which iGAS infections in children fulfil the diagnostic criteria for STSS is lower than that in adults [15]. Consequently, it has been highlighted that the current surveillance system in Japan may not adequately reflect the actual burden of severe GAS infections in children. In this context, a comprehensive epidemiological survey of severe paediatric GAS infections in Japan is warranted.

In this study, we examined the clinical characteristics and outcomes of severe paediatric GAS infections in Japan, as well as the changes in epidemiological trends before and after the COVID-19 pandemic.

## Methods

### Study design and data sources

We conducted a multicentre retrospective study across the educational training programme hospitals of the Japanese Society for Paediatric Infectious Diseases (JSPID). These 86 hospitals are geographically dispersed throughout Japan (Supplementary Figure 1), with 41 being designated as “core hospital paediatric departments” by the Japanese Paediatric Society, accounting for approximately one-third (41/119) of the total nationwide [16]. Data were collected regarding paediatric patients admitted to these hospitals with severe GAS infections between 1 January 2019 and 31 March 2024. A two-phase questionnaire-based survey was conducted. In the initial phase, the participating hospitals were asked whether severe GAS infections had been encountered at their institution. Hospitals reporting eligible cases were requested to complete the second phase, wherein detailed clinical information was extracted from electronic medical records. The number of cases, infection diagnoses, treatments, and outcomes were compared between pre-COVID-19 (April 2019–March 2020) and post-COVID-19 pandemic (April 2023–March 2024) periods.

### Case definition and clinical data

Eligible cases were defined as children under 18 years of age admitted owing to severe GAS infection. In this study, severe GAS infection was defined as a case meeting at least one of the following criteria: confirmed and probable cases according to the iGAS infection surveillance guidelines [17].

1. Cases in which GAS was detected by culture or nucleic acid test from a sterile site (e.g., blood, cerebrospinal fluid, or pleural fluid).
2. Cases of clinically severe infection (e.g., septic shock, STSS, and necrotizing fasciitis) in which GAS was detected in non-sterile sites, and no other bacterial aetiology was identified.

The second phase collected data on patient demographics, symptoms, medical history, culture results, infection diagnoses, treatments, outcomes, and STSS notification status. Underlying conditions were defined as pre-existing chronic medical conditions documented in the medical records before disease onset, including congenital heart disease, chronic lung disease, prematurity, atopic dermatitis, neurologic disorders, genetic syndromes, malignancy, and immunodeficiency.

### Statistical analysis

Categorical variables are summarized as counts (%) and continuous variables as medians with interquartile ranges (IQRs). For between-group comparisons, categorical variables were analysed for associations with clinical characteristics, severity, and outcomes, before and after the COVID-19 pandemic. Fisher’s exact test was used for univariate risk ratios and 95% confidence intervals, whereas continuous variables were analysed for *p*-values using the Wilcoxon rank sum test. All *p*-values were two-sided, and *p* < 0.05 was considered statistically significant. All statistical analyses were conducted using the JMP Pro V18 Statistical Software (SAS Institute Inc., Cary, NC, USA).

### Ethical considerations

The study was approved by the Ethics Committee of Chiba University Medical Mycology Research Centre (Approval No. 24–36).

## Results

In the initial survey, 94% (81/86) of eligible hospitals responded. The second phase of the survey included 27 hospitals that reported relevant cases in the initial survey and obtained a 100% response rate. Data from 90 paediatric patients with severe GAS infections were collected. After excluding five cases out of the study period, one recurrent case (counted as a single case), and one outpatient case with an unclear course, 83 cases were included in the final analysis (Supplementary Figure 2). Although the case definition used for this collection included clinically severe infections in which GAS was detected in non-sterile sites, all collected cases in this study involved GAS detection in sterile sites.

**Figure 1.**
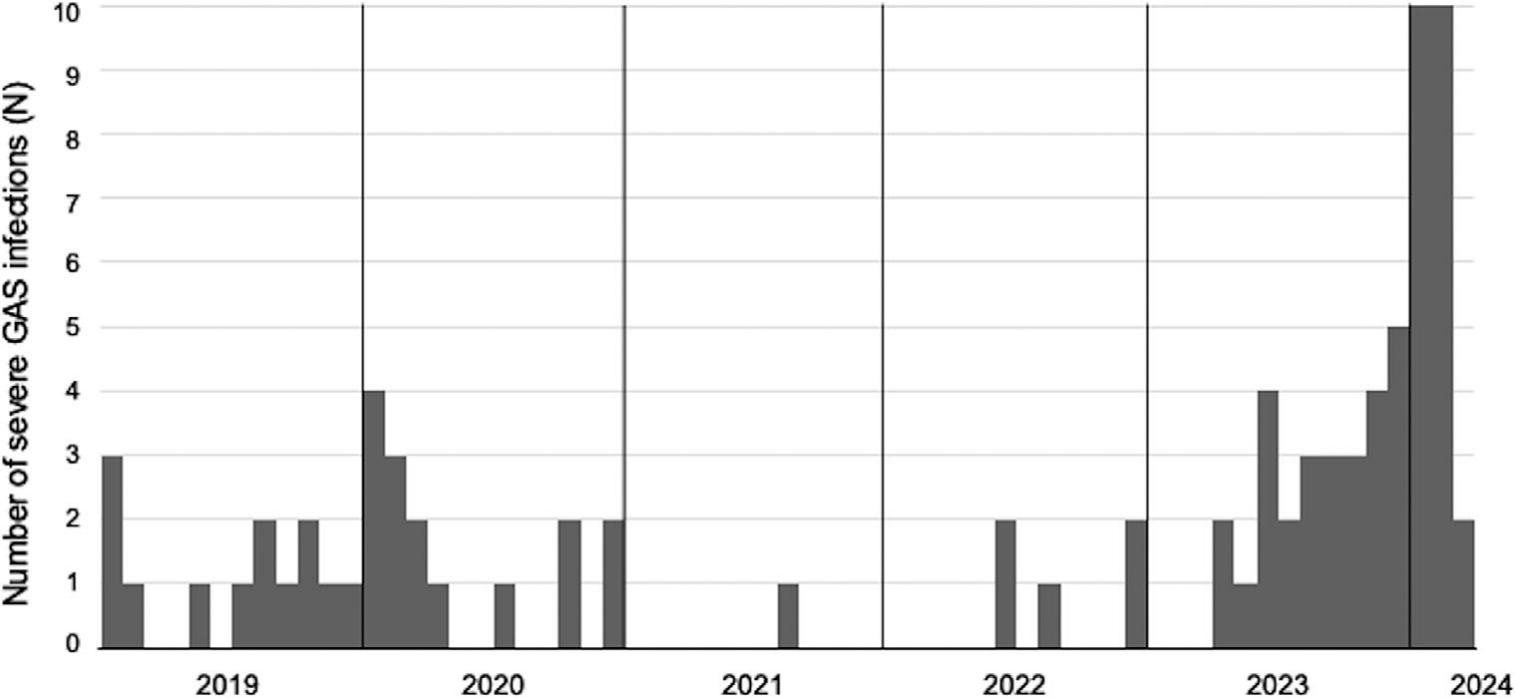
Monthly number of severe Group A Streptococcus infections among individuals below 18 years of age, January 2019–March 2024 (n = 83). *Note*: The number of cases declined during the COVID-19 pandemic, but increased in the summer of 2023, peaking between January and February 2024.

The monthly trends for these cases are depicted in Figure 1. The number of cases declined during the COVID-19 pandemic, but began to increase in the summer of 2023, peaking in January–February 2024 within the observation period. The demographic characteristics of patients are summarized in Table 1. The median age was 4 (IQR: 1–8) years. Patients under 3 years of age accounted for 43% of cases, with 1-year-olds being the most prevalent (Supplementary Figure 3). In STSS and necrotizing fasciitis cases, the median age was 9 (IQR: 3.8–13.3) years and 8 (IQR: 2.5–9) years, respectively (Supplementary Table 1). Males comprised 52% (43/83) of cases, and 54% (45/83) had no underlying conditions. During the study period, the highest number of cases was reported in Kanto, central Japan, whereas no cases were reported in Hokkaido, northern Japan.

**Table 1.** Key demographic characteristics of patients with severe Group A Streptococcus infections admitted to the educational training programme hospitals of JSPID, January 2019–March 2024

	*N* (%) or median (IQR) *N* = 83
Patient characteristics	
Age (years)	4 (1–8)
Male sex, *n* (%)	43 (52)
Underlying medical condition, *n* (%)	38 (46)
Regions, *n* (%)	
Hokkaido	0 (0)
Tohoku	1 (1)
Kanto	47 (57)
Chubu	9 (11)
Kinki	11 (13)
Chugoku, Shikoku	1 (1)
Kyushu, Okinawa	14 (17)

IQR, interquartile range; JSPID, Japanese Society for Paediatric Infectious Diseases.

### Clinical presentations

The most prevalent clinical symptom was fever (93%, 77/83) (Supplementary Table 2). 20% (17/83) reported a history of respiratory viral infection within 1 month preceding hospitalization (Table 2). No patients had a history of varicella-zoster virus infection, and 6% (5/83) had a prior diagnosis of GAS pharyngitis.

**Table 2.** Clinical presentations of patients with severe Group A Streptococcus infections admitted to the educational training programme hospitals of JSPID, January 2019–March 2024

	*N* = 83
Associated clinical features in the month preceding admission, *n* (%)	
Respiratory viral infection^a^	17 (20)
Pharyngitis symptoms	5 (6)
Trauma	3 (4)
Surgery	1 (1)
Varicella zoster virus infection	0 (0)
Clinical manifestations^b^, *n* (%)	
Pneumonia	21 (25)
With Empyema	16 (19)
Bone and joint infections	20 (24)
Bacteraemia without focus	16 (19)
Skin and soft-tissue Infection	12 (14)
Streptococcal toxic shock syndrome	10 (12)
Pyomyositis/muscle abscess	5 (6)
Necrotizing fasciitis	4 (5)
Infective endocarditis	3 (4)
Surgical site infection	3 (4)
Lymphadenitis	2 (2)
Other^c^	8 (10)

IQR, interquartile range; RADT, rapid antigen detection test.

a Nine cases of influenza, three cases of human metapneumovirus, two cases of respiratory syncytial virus, two cases of entero/rhinovirus, one case of SARS-CoV-2, and one case of adenovirus.

b Some patients presented with more than one clinical manifestation.

c One case each of meningitis, brain abscess, infected aneurysm, catheter-related bloodstream infection, parapharyngeal space abscess, pharyngitis, parotid gland injury, and lymphangioma infection.

The most frequently detected site for GAS was blood (76%, 63/83), followed by pleural fluid (19%, 16/83), tissue (10%, 8/83), and joint fluids (7%, 6/83). GAS was detected in the pharynx in 5% (4/83) of cases. Nearly all cases (99%, 82/83) were diagnosed through culture testing, except for one empyema case, which was culture-negative and diagnosed using a pleural fluid nucleic acid test. In four cases (5%), rapid antigen testing detected GAS in non-pharyngeal specimens.

The most prevalent clinical manifestation was pneumonia (25%, 21/83), of which 76% (16/21) were complicated by empyema. Bone and joint infections accounted for 24% (20/83), bacteraemia without focus accounted for 19% (16/83), and skin and soft tissue infections accounted for 14% (12/83). The diagnostic criteria for STSS were met in 12% of cases (10/83).

### Treatment and outcomes

Initial treatment with β-lactam antibiotics was administered in 98% (79/83) of cases (Table 3). Antimicrobial de-escalation to single targeted β-lactam therapy, such as ampicillin, amoxicillin, cefazolin, cephalexin, or penicillin G, was achieved in 80% (64/83) of cases. Transition to oral antimicrobials was achieved in 65% of patients (52/83). The median duration of total antibiotic therapy was 22 (IQR, 14–43) days, with a median intravenous duration of 15 (IQR, 14–25) days. As adjunctive therapy, intravenous immunoglobulin (IVIG) was administered in 4% (3/83) of cases, and clindamycin combination therapy was administered in 24% (19/83) of cases. Clindamycin was administered most frequently for STSS (75%, 6/8), necrotizing fasciitis (75%, 3/4), and empyema (44%, 7/16) (Supplementary Table 1). Surgical intervention was performed in 45% (38/83) of cases, more commonly in necrotizing fasciitis (100%, 4/4) and empyema (94%, 15/16).

**Table 3.** Treatment and outcomes of patients with severe Group A Streptococcus infections admitted to the educational training programme hospitals of JSPID, January 2019–March 2024

	*N* (%) or Median (IQR)
Treatment, *n* (%)	*N* = 80^a^
β-lactam antibiotic use during initial treatment	78 (98)
De-escalation to single narrow-spectrum β-lactam antibiotics^b^	64 (80)
Transition to oral antibiotics	52 (65)
Clindamycin use	18 (23)
Clindamycin duration (days)	7.5 (4.3–13)
Duration of intravenous therapy (days)	15 (12.5–25.8)
Duration of total therapy (days)	21.5 (14–43.5)
Intravenous immunoglobulin use	3 (4)
Surgical intervention	38 (48)
Invasive ventilation	18 (23)
Non-invasive ventilation	2 (3)
Vasoactive drug use	10 (13)
Outcomes, *n* (%)	*N* = 82^c^
ICU admission	33 (40)
ICU LOS (days)	6 (3–11)
Hospital LOS (days)	17.5 (14–35.5)
Presence of sequelae	7 (9)
30-day mortality	3 (4)

ICU, intensive care unit; IQR, interquartile range; LOS, length of stay.

a Excludes two cases of death on the same or the day following admission without antimicrobial therapy, and one case of transfer within 24 h of admission.

b Ampicillin, amoxicillin, cefazolin, cephalexin, or penicillin G.

c Excludes one case of transfer within 24 h of admission.

Intensive care unit (ICU) admission was required in 40% (33/83) of cases, with higher rates among empyema (100%, 16/16), STSS (80%, 8/10), and necrotizing fasciitis (75%, 3/4) cases (Table 3 and Supplementary Table 1). Invasive mechanical ventilation and vasoactive drugs were required in 23% (19/83) and 13% (11/83) of patients, respectively. The median length of hospital stay was 17.5 (IQR, 14–35.5) days. Three cases (4%) were fatal, all within 2 days of admission or symptom onset. Two patients died before antibiotic administration was initiated. The three fatal cases shared common characteristics: bacteraemia without focus, fulfilment of the STSS criteria, and underlying malignancies. NESID received 17% (14/83) of the reports of patients with STSS. Four of these cases were retrospectively determined as not meeting the STSS criteria.

### Comparison before and after the COVID-19 pandemic

Comparing the year preceding the COVID-19 pandemic (April 2019–March 2020) to the year following the pandemic (April 2023–March 2024), the number of cases of severe paediatric GAS infection increased 2.7-fold (n = 18 pre-pandemic, mean cases per month: 1.5; n = 49 post-pandemic, mean cases per month: 4.1) (Supplementary Table 3). No statistically significant differences were observed in the clinical characteristics, treatment, or outcomes between the two periods.

## Discussion

To our knowledge, this study constitutes the first multicentre nationwide hospital-based survey of severe paediatric GAS infections in Japan. Our findings indicate that the number of severe paediatric GAS infections increased from the summer of 2023, surpassing pre-COVID-19 pandemic levels. This trend is consistent with variations in GAS pharyngitis and STSS documented in Japan’s national surveillance system [14]. Three primary factors may have contributed to this increase. First, infection control measures during the pandemic likely reduced exposure to GAS, resulting in a decline in herd immunity. A study of ten European countries reported a significant reduction in serum antibodies to GAS after the COVID-19 pandemic, coinciding with the increase in iGAS infections among children following the COVID-19 pandemic in Europe [18]. In Japan, a comparable surge was observed approximately one year later than in Western countries [19]. This delay may be attributed to the more cautious approach adopted by the population in easing infection control measures compared with the more immediate lifting of measures in other countries. Furthermore, the change in the COVID-19 status under the Infectious Disease Control Law in May 2023 may have facilitated psychological and social deregulation.

Second, the prevalence of respiratory viral infections such as influenza may have heightened the risk of secondary bacterial infections. Respiratory viral infections are considered risk factors for iGAS. Several studies on paediatric iGAS infections after the COVID-19 pandemic have reported a high rate of concurrent infections with respiratory viruses and a correlation with the timing of epidemics [5, 19–21]. Surveillance data on respiratory viral infections in Japan during the study period revealed that influenza was epidemic from late 2023 to early 2024, coinciding with the epidemic period of severe GAS infections. In contrast, respiratory syncytial virus and SARS-CoV-2 did not coincide with the epidemic of severe GAS infections. Therefore, although some viral respiratory infections may have contributed to the increase in severe GAS infections, the current increase may not be solely attributable to this factor.

Third, an increase in the proportion and number of *Streptococcus pyogenes* M1UK strains, recognized for high virulence and transmissibility, may have contributed to the observed trends. Some European studies have proposed that the increase in iGAS cases is related to the spread of M1UK [2, 22–24]. In contrast, other studies have reported a shift in epidemic strains to types other than M1UK. Consequently, some perspectives refute an epidemiological link with the emergence of any specific strains, and no global consensus on this issue exists [8, 9, 21, 25, 26]. In Japan, there has been a reported increase in the number and proportion of M1UK strains among patients with STSS, particularly in metropolitan and surrounding areas [27]. However, the association of these strains with increased STSS and GAS pharyngitis remains unclear. As this study did not include bacterial strain collection and analysis, the relationship between the increase in severe paediatric GAS cases and strain distribution remains unknown and warrants further investigation. The epidemiological trends of iGAS infections after 2023 vary across countries. Therefore, ongoing surveillance of iGAS infection trends and epidemiological characteristics is imperative in Japan.

Our multicentre survey also identified regional disparities. The Kanto region reported the highest number of patients, whereas no cases were reported in Hokkaido during the survey period. This survey encompassed 14 hospitals in Hokkaido, including three university hospitals and other major hospitals, minimizing the likelihood of overlooked cases. Hokkaido is unique in that it is the northernmost prefecture in Japan, has a low population density, and is surrounded by the sea. The absence of applicable cases may be attributable to regional characteristics or prevalent strains differing from those in other regions. However, this survey did not elucidate these details, and additional studies are required.

The median age of patients in this study was 4 years, consistent with the median age range of 3–5 years reported in international studies on paediatric iGAS [5, 7, 8, 21, 28, 29]. Although an increase in the number of iGAS infections in individuals younger than 5 years has been emphasized in other nations after the pandemic [19], no significant change in the age distribution was identified before or after the pandemic in the present study. GAS pharyngitis is more common among school-aged children and adolescents, with a peak incidence at 7 to 8 years of age. In children under 3 years, the probability of GAS pharyngitis is low, and testing is generally not recommended [15]. In contrast to GAS pharyngitis, iGAS infections are more frequently observed in younger age groups, and paediatricians should be cognisant of the differences in age distribution between GAS pharyngitis and iGAS infection. In the present study, pharyngitis was only diagnosed before the onset of severe GAS infection in 6% of cases. Although symptomatic GAS pharyngitis rarely precedes iGAS infection [14, 28], it remains uncertain whether invasive infection results from unrecognized noninvasive infection or colonization [29, 30]. The results of this study indicate that symptomatic GAS pharyngitis and severe GAS infection have distinct characteristics and risk factors, underscoring the challenges related to early diagnosis of iGAS infection.

The most prevalent clinical manifestations observed in this study were pneumonia/empyema with bone and joint infections, bacteraemia without focus, and skin and soft tissue infections. Several studies on paediatric iGAS infections post-pandemic have reported higher rates of pneumonia and increased severity [8, 19, 21]. Although we did not identify statistically significant differences in clinical manifestations or disease severity pre- and post-pandemic, pneumonia/empyema cases consistently exhibited high severity, requiring ICU admission, mechanical ventilation, and surgical intervention more frequently than other infection types. Among the clinical presentations of paediatric iGAS infections, pneumonia/empyema is considered particularly severe, alongside STSS and necrotizing fasciitis [21, 29]. GAS is the major causative agent of pneumonia/empyema in children, and previous studies have reported higher disease severity compared to that caused by other pathogens, necessitating longer hospital stays and a greater need for drainage procedures [31, 32]. Furthermore, paediatric iGAS infections are associated with a higher proportion of pneumonia cases and empyema complications than adult iGAS infections [4]. These findings underscore the significance of pneumonia/empyema in the clinical presentation of severe paediatric GAS. In this study, 12% of patients met the diagnostic criteria for STSS. Among these STSS cases, 30% died within 2 days of hospitalization or symptom onset, and all were patients with underlying malignancies. Compared with adults, the incidence of STSS in children with iGAS infection is reportedly low, ranging from less than 5% to 10% [15, 21, 29, 33, 34]. The prognosis of paediatric STSS is also observed to be more favourable than that of adults; however, mortality rates vary widely, ranging from 4% to 40% depending on the report [4, 33, 35, 36]. Although malignancy is considered a risk factor for mortality in STSS, a previous study has reported that STSS can result in serious outcomes even in otherwise healthy children [34, 35]. Large-scale reports on paediatric STSS are limited, and further studies are required to better elucidate the associated risk factors for mortality.

In most cases in this study, β-lactams were the initial treatment of choice, with their coverage depending on the clinical presentation. Despite the severity of the cases, the majority of patients were ultimately transitioned to single targeted β-lactam therapy, and more than half were switched to oral antibiotics. Both IVIG and clindamycin combination therapies have been documented to enhance the prognosis of severe GAS infections, particularly necrotizing fasciitis and STSS [37]. However, these therapies were employed in only 4% and 24% of cases, respectively. Clindamycin is often administered in conjunction with penicillin owing to its ability to inhibit exotoxin and M-protein production, as well as the eagle effect [38, 39]. Its efficacy has also been evidenced in iGAS infections beyond necrotizing fasciitis and STSS [40, 41]. Reports on these combination therapies have mainly focused on adult cases, and the current findings suggest that they may not be sufficiently prevalent in paediatric clinical settings in Japan. However, no clear evidence that adjunctive clindamycin is ineffective or harmful in children is established, and its use appears reasonable in paediatric patients as well.

The length of hospital stay observed in this study was longer than that reported in studies from Western countries [8, 9, 29]. Given the duration of intravenous therapy in this study, patients were likely discharged after completing intravenous therapy and several days of inpatient monitoring. This practice is consistent with the medical situation in Japan, where hospital stays tend to be longer owing to factors including universal health insurance and low copayments, the limited availability of outpatient parenteral antimicrobial therapy and post-acute home or facility care, and a cultural tendency to confirm clinical stability after completing intravenous therapy before discharge.

High response rates and the substantial number of cases collected are strengths of this study. However, the study had some limitations. First, it only included hospitals from 21 of Japan’s 47 prefectures, raising concerns regarding representativeness. Nevertheless, the hospitals surveyed were widely distributed, primarily in the more populated regions of Japan. Second, this study was limited to data collection based on questionnaires, precluding the possibility of conducting strain analysis or evaluating bacteriological characteristics and their changes. Third, the comparison of data pre- and post-COVID-19 may not have yielded statistically significant between-group differences regarding the clinical characteristics, treatment, and outcomes owing to the limited number of cases before the pandemic. Furthermore, the comparison before and after this pandemic only contrasts the absolute number of patients, and does not allow for comparing relative numbers such as the proportion of hospitalized patients or incidence rates. However, based on rough estimates of annual hospitalization trends, the number of hospitalized paediatric patients decreased from 2020 to 2022, but no significant difference was observed between 2019 and 2023 and beyond, suggesting that the number of paediatric patients with severe GAS infection likely increased relatively as well.

In summary, this is the first multicentre nationwide hospital-based study of severe paediatric GAS infections in Japan and may serve as a vital reference for future epidemiological comparisons both domestically and internationally. The survey revealed an increased number of paediatric patients with severe GAS infections in Japan following the COVID-19 pandemic. Our data suggest that many severe paediatric GAS cases, such as pneumonia/empyema, do not meet the STSS reporting criteria. This highlights the limitations of the current surveillance system in Japan, which only covers STSS, in accurately capturing the true burden of severe paediatric GAS infections.

## Supporting information

10.1017/S0950268826101162.sm001 Tada et al. supplementary material Tada et al. supplementary material

## Data Availability

Data are available upon reasonable request to the corresponding author.

## Supplementary material

The supplementary material for this article can be found at http://doi.org/10.1017/S0950268826101162.
