# Supplementary material for: Clinical features and trends of severe paediatric group A streptococcal infections in Japan in the post-COVID-19 pandemic era
Source: Epidemiol Infect. 2026 Feb 19;154:e35. doi: 10.1017/S0950268826101162 (PMC13100923; doi:10.1017/S0950268826101162)
Supplement: Tada et al. supplementary material [file S0950268826101162sup001.docx]

**Supplementary Tables and Figures** **- Clinical Features and Trends of Severe Pediatric Group A Streptococcal Infections in Japan in the Post-COVID-19 Pandemic Era**

Contents

[Supplementary Table 1. Clinical features, treatment, and outcomes of patients with severe GAS infections admitted to the educational training program hospitals of the JSPID, January 2019 to March 2024 according to infection diagnosis 2](#_Toc220328868)

[Supplementary Table 2. Clinical symptoms and laboratory findings of patients with severe GAS infections admitted to the educational training program hospitals of the JSPID, January 2019 to March 2024 4](#_Toc220328869)

[Supplementary Table 3. Clinical features, treatment, and outcomes of patients with severe GAS infections admitted to the educational training program hospitals of the JSPID, before and after the COVID-19 pandemic, January 2019 to March 2024 6](#_Toc220328870)

[Supplementary Table 4. List of JSPID education and training program hospitals by region 8](#_Toc220328871)

[Supplementary Figure 1. Distribution and number of education and training program hospitals of the JSPID by region 10](#_Toc220328872)

[Supplementary Figure 2. Flowchart of patient enrollment in the first and second surveys 11](#_Toc220328873)

[Supplementary Figure 3. Age distribution of eligible patients with severe GAS infections admitted to the educational training program hospitals of the JSPID, January 2019 to March 2024 12](#_Toc220328874)

# **Supplementary Table 1. Clinical features, treatment, and outcomes of patients with severe GAS infections admitted to the educational training program hospitals of the JSPID, January 2019 to March 2024 according to infection diagnosis**

|  | N (%) or Median (IQR) | | | | | | |
| --- | --- | --- | --- | --- | --- | --- | --- |
|  | Pneumonia | | Bone and joint infections | Bacteremia without focus | Skin and soft-tissue infection | Streptococcal toxic shock syndrome | Necrotizing fasciitis |
|  | All | With empyema |  |  |  |  |  |
| **Characteristic**  Age (years)  Respiratory viral infection, n (%) | N=21  2 (1–9)  8 (38) | N=16  2 (1–9)  7 (44) | N=20  4 (2–6)  1 (5) | N=16  4 (1.5–8)  2 (13) | N=12  6 (2–8.8)  3 (25) | N=10  9 (3.8–13.3)  1 (10) | N=4  8 (2.5–9)  0 (0) |
| **Treatment,** n (%)  Narrowest spectrum β-lactam  Ampicillin or amoxicillin  Ampicillin-sulbactam or amoxicillin-clavulanate  Cefazolin or cephalexin  Penicillin G  Transition to oral antibiotics  Clindamycin use  Surgical intervention  Intravenous immunoglobulin use  Duration of total therapy (days) | N=21  18 (86)  1 (5)  0 (0)  0 (0)  16 (76)  8 (38)  16 (76)  1 (5)  30 (21–56.5) | N=16  14 (88)  1 (6)  0 (0)  0 (0)  13 (81)  7 (44)  15 (94)  1 (6)  30.5 (22.8–58.5) | N=20  16 (80)  0 (0)  2 (10)  2 (10)  17 (85)  2 (10)  10 (50)  0 (0)  42 (29.3–86) | N=13^a^  10 (77)  0 (0)  1 (8)  0 (0)  8 (63)  1 (8)  0 (0)  0 (0)  14 (13–15) | N=12  5 (42)  1 (8)  3 (25)  0 (0)  5 (42)  2 (17)  3 (25)  0 (0)  14 (14–20.5) | N=8^c^  2 (25)  0 (0)  1 (13)  2 (25)  4 (50)  6 (75)  7 (88)  2 (25)  44.5 (18–49) | N=4  3 (75)  0 (0)  0 (0)  2 (50)  1 (25)  3 (75)  4 (100)  1 (25)  49 (46–82.8) |
| **Outcomes,** n (%)  ICU admission  Invasive ventilation  Vasoactive drugs use  Death  Hospital LOS (days) | N=21  20 (95)  12 (57)  6 (29)  0 (0)  27 (17–40) | N=16  16 (100)  9 (56)  4 (25)  0 (0)  29 (19.8–40.5) | N=20  3 (15)  1 (5)  1 (5)  0 (0)  18 (15–34) | N=15^b^  1 (7)  1 (7)  1 (7)  3 (20)  13 (7.5–14) | N=12  3 (25)  2 (17)  0 (0)  0 (0)  16 (11–22) | N=10  8 (80)  7 (70)  6 (60)  3 (30)  34 (4–63.3) | N=4  3 (75)  3 (75)  3 (75)  0 (0)  68.5 (40.8–88.8) |

IQR, interquartile range; ICU, intensive care unit; LOS, length of stay

^a^Excludes one case that was transferred within 24 h after admission and two cases of death on the same day or the day after admission without antimicrobial therapy

^b^Excludes one case that was transferred within 24 h after admission

^c^Excludes two cases of death on the same day or the day after admission without antimicrobial therapy

GAS, Group A *Streptococcus*; ICU, intensive care unit; JSPID**,** Japanese Society for Pediatric Infectious Diseases; LOS, length of stay

# **Supplementary Table 2. Clinical symptoms and laboratory findings of patients with severe GAS infections admitted to the educational training program hospitals of the JSPID, January 2019 to March 2024^a^**

|  | N=83 |
| --- | --- |
| **Symptoms**, n(%) |  |
| Fever | 77 (93) |
| Localized redness, swelling, severe pain | 33 (40) |
| Cough, wheezing | 18 (21) |
| Arthralgia | 13 (15) |
| Central nerve system symptoms (seizure, loss of consciousness) | 11 (13) |
| Sore throat | 8 (10) |
| Hypotension | 6 (7) |
| Abdominal pain, vomiting, diarrhoea | 5 (6) |
| Generalized erythema | 4 (5) |
| Respiratory distress, labored breathing | 4 (5) |
| Chest pain | 2 (2) |
| **Sites of GAS isolation,** n (%) |  |
| Blood | 63 (76) |
| Pleural fluid | 16 (19) |
| Tissue | 8 (10) |
| Joint fluid | 6 (7) |
| Wound | 4 (5) |
| Pharynx | 4 (5) |
| Sputum | 3 (4) |
| Bone | 2 (2) |
| Pus | 2 (2) |
| Cerebrospinal fluid | 1 (1) |
| Verrucous tumor | 1 (1) |
| Urine | 1 (1) |
| **Methods of GAS isolation,** n (%) |  |
| Culture | 82 (99) |
| Nucleic acid test | 2 (2)^b^ |
| Rapid antigen detection test | 8 (10)^c^ |

^a^ Some patients were included in more than one category

^b^ One case of blood and one case of pleural fluid

^c^ Four cases of pharynx, two cases of pleural pus, one case of joint fluid, and one case of pleural fluid

GAS, Group A *Streptococcus*; JSPID**,** Japanese Society for Pediatric Infectious Diseases

# **Supplementary Table 3. Clinical features, treatment, and outcomes of patients with severe GAS infections admitted to the educational training program hospitals of the JSPID, before and after the COVID-19 pandemic, January 2019 to March 2024**

|  | Pre-pandemic  N=18 | Post-pandemic  N=49 | RR (95%CI) / *p*-value |
| --- | --- | --- | --- |
| **Characteristic** |  |  |  |
| Median age  Respiratory viral infection | 4 (1–8)  1 (5%) | 5 (2–9)  13 (17%) | 0.37  4.78 (0.67–33.93) |
| **Clinical manifestations^a^** |  |  |  |
| Pneumonia  　 With empyema  　Bone and joint infections  　Bacteremia without focus  　Skin and Soft-tissue Infection  　Streptococcal toxic shock syndrome  　Necrotizing fasciitis | 4 (22%)  3 (17%)  7 (39%)  4 (22%)  2 (11%)  2 (11%)  1 (6%) | 14 (29%)  12 (24%)  10 (20%)  8 (16%)  7 (14%)  6 (12%)  3 (6%) | 1.29 (0.49–3.40)  1.47 (0.47–4.61)  0.52 (0.24–1.17)  0.73 (0.25–2.15)  0.96 (0.79–1.18)  1.10 (0.24–4.97)  0.99 (0.87–1.14) |
| **Treatment** |  |  |  |
| Clindamycin use | 2 (11%) | 15 (31%) | 2.76 (0.70–10.87) |
| Surgical intervention | 6 (33%) | 25 (51%) | 1.56 (0.77–3.17) |
| **Outcome** |  |  |  |
| ICU admission | 7 (39%) | 20 (26%) | 1.07 (0.55–2.09) |
| Death | 1 (6%) | 1 (2%) | 0.38 (0.025–5.68) |
| Hospital LOS (days) | 17.5 (14.8–25) | 18.5 (14–38.5) | 0.42 |

To compare the two groups before and after COVID-19, categorical variables were analyzed for associations with clinical characteristics, treatment, and outcome using univariate risk ratios (RR) and 95% confidence intervals (CIs) using Fisher's exact test, and p-values for continuous variables were analyzed using the Wilcoxon rank sum test. All p-values were two-tailed, and p <0.05 was considered statistically significant.

**^a^**Patients may present with more than one clinical manifestation

JSPID**,** Japanese Society for Pediatric Infectious Diseases; IQR, interquartile range; ICU, intensive care unit; LOS, length of stay

# **Supplementary Table 4. List of JSPID education and training program hospitals by region**

| Regions (N=86) | Education and training program hospitals |
| --- | --- |
| Hokkaido (n=14) | Hokkaido University Hospital, Sapporo City General Hospital, Oji General Hospital, KKR Sapporo Medical Center, Japanese Red Cross Kitami Hospital, Teine Keijinkai Hospital, Sapporo Medical University Hospital, Asahikawa Medical University Hospital, Hokkaido Medical Center for Child Health and Rehabilitation, Asahikawa Kosei Hospital, Nayoro City General Hospital, Furano Kyokai Hospital, Abashiri Kousei Hospital, Engaru Kosei Hospital |
| Tohoku (n=6) | Fukushima Medical University Hospital, Hoshi General Hospital, Soma General Hospital, Miyagi Children’s Hospital, Tohoku University Hospital, Tohoku Rosai Hospital |
| Kanto (n=30) | Tokyo Metropolitan Children’s Medical Center, National Center for Child Health and Development, Nihon University Itabashi Hospital, Chiba Children's Hospital, Chiba University Hospital, Chiba Kaihin Municipal Hospital, Tokyo Women’s University Yachiyo Medical Center, Teikyo University Chiba Medical Center, Saitama Children’s Medical Center, Keio University Hospital, Kawasaki Municipal Hospital, NHO Tochigi Medical Center, Saitama City Hospital, Tokyo Metropolitan Ohtsuka Hospital, National Hospital Organization Saitama Hospital, National Hospital Organization Tokyo Medical Center, Yokohama Municipal Citizen's Hospital, Subaru Health Insurance Society Ota Memorial Hospital, Juntendo University Hospital, Juntendo University Urayasu Hospital, San-Ikukai Hospital, Juntendo University Nerima Hospital, St. Marianna University Hospital, St. Marianna University Yokohama City West Hospital, Kawasaki Municipal Tama Hospital, Dokkyo Medical University Hospital, Gunma Children's Medical Center, Fujisawa City Hospital, Yokohama City University Medical Center, Kanagawa Children's Medical Center, |
| Chubu (n=11) | Niigata University Medical & Dental Hospital, Niigata City General Hospital, Aichi Children’s Health and Medicine Center, Nagoya University Hospital, Konan Kosei Hospital, Nagano Children's Hospital, Fujita Health University School of Medicine, Kariya Toyota General Hospital, Toyokawa City Hospital, Kanazawa University Hospital, NHO Kanazawa Medical Center |
| Kinki (n=12) | NHO Mie National Hospital, Mie University Hospital, Hyogo Prefectural Kobe Children's Hospital, Hyogo Prefectural Amagasaki General Medical Center, Nara Medical University, Nara Prefecture General Medical Center, Japan Community Health care Organization Hoshigaoka Medical Center, Nara Prefectural Seiwa Medical Center, Yao Municipal Hospital, Yamato Takada City Hospital, Tazuke Kofukai Medical Research Institute, Kitano Hospital, Osaka City General Hospital |
| Chugoku, Shikoku  (n=6) | Kawasaki Medical School Hospital, Okayama University Hospital, Kurashiki Central Hospital, Kawasaki Medical School General Medical Center, Ehime University Hospital, Ehime Prefectural Niihama Hospital |
| Kyushu, Okinawa (n=7) | Fukuoka Children's Hospital, Kyushu University Hospital, Kurume University Hospital, Fukuoka University Hospital, Hospital of the University of Occupational and Environmental Health, Okinawa Prefectural Nanbu Medical Center & Children's Medical Center, Okinawa Prefectural Chubu Hospital |

JSPID**,** Japanese Society for Pediatric Infectious Diseases

# **Supplementary Figure 1. Distribution and number of education and training program hospitals of the JSPID by region**


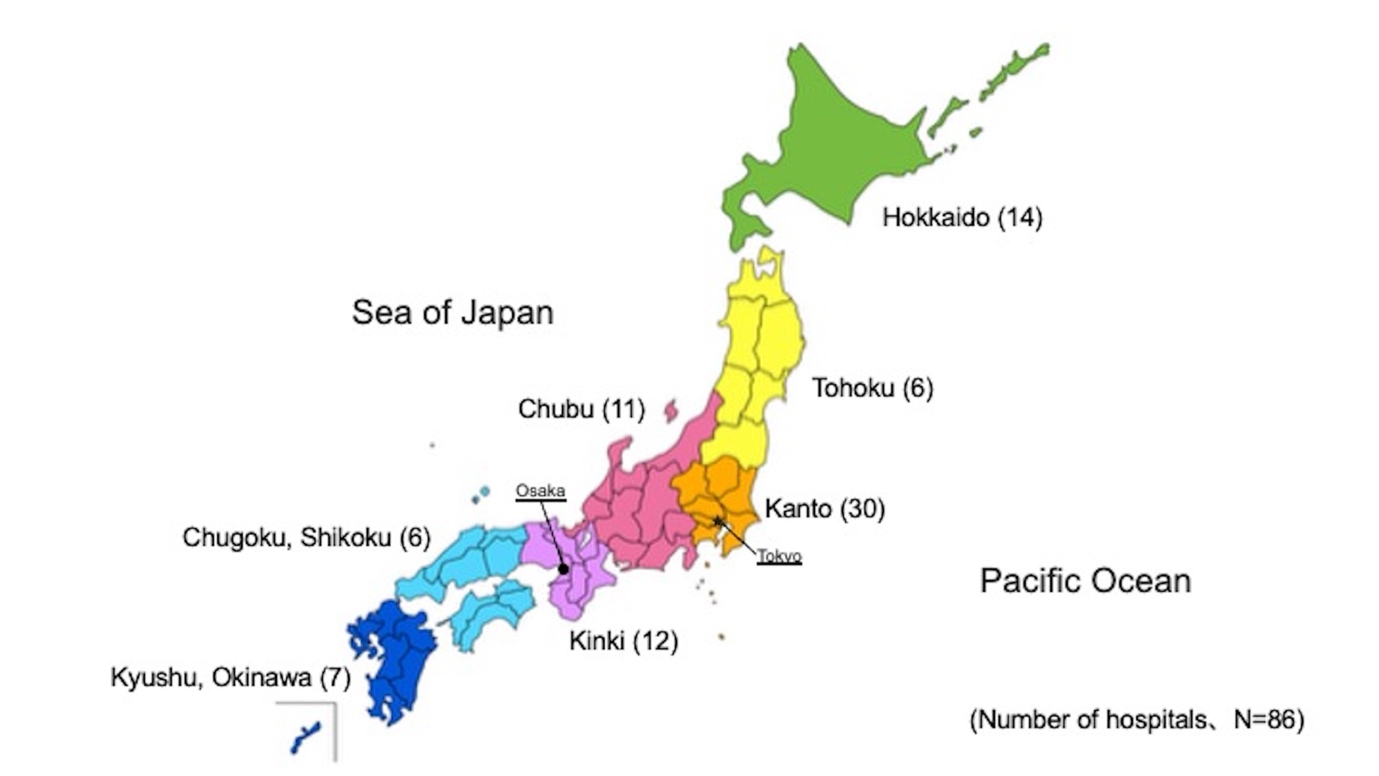
A total of 86 hospitals covering major regions widely distributed across Japan.

JSPID**,** Japanese Society for Pediatric Infectious Diseases

# **Supplementary Figure 2. Flowchart of patient enrollment in the first and second surveys**


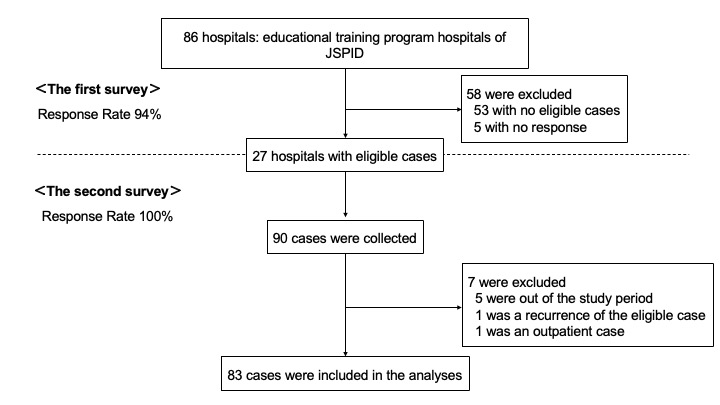


The response rates for the first and second surveys were 94% and 100%, respectively. Finally, 83 cases of severe pediatric GAS infections were analyzed.

GAS, Group A *Streptococcus*; JSPID**,** Japanese Society for Pediatric Infectious Diseases

# **Supplementary Figure 3. Age distribution of eligible patients with severe GAS infections admitted to the educational training program hospitals of the JSPID, January 2019 to March 2024**


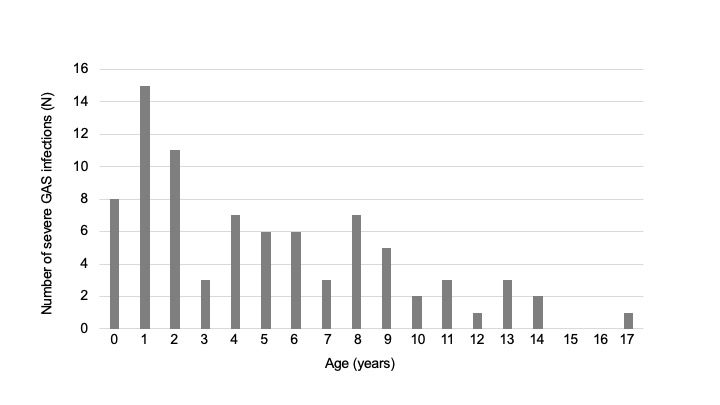


The number of patients tended to be higher at younger ages, with the highest number of patients being one-year-old.

GAS, Group A *Streptococcus;* JSPID**,** Japanese Society for Pediatric Infectious Diseases
